# Effects of Increased Nitrogen Deposition and Precipitation on Seed and Seedling Production of *Potentilla tanacetifolia* in a Temperate Steppe Ecosystem

**DOI:** 10.1371/journal.pone.0028601

**Published:** 2011-12-14

**Authors:** Yang Li, Haijun Yang, Jianyang Xia, Wenhao Zhang, Shiqiang Wan, Linghao Li

**Affiliations:** 1 State Key Laboratory of Vegetation and Environmental Change, Institute of Botany, Chinese Academy of Sciences, Xiangshan, Beijing, China; 2 Graduate School of Chinese Academy of Sciences, Yuquanlu, Beijing, China; 3 Key Laboratory of Plant Stress Biology, College of Life Sciences, Henan University, Kaifeng, Henan, China; Trinity College Dublin, Ireland

## Abstract

**Background:**

The responses of plant seeds and seedlings to changing atmospheric nitrogen (N) deposition and precipitation regimes determine plant population dynamics and community composition under global change.

**Methodology/Principal Findings:**

In a temperate steppe in northern China, seeds of *P. tanacetifolia* were collected from a field-based experiment with N addition and increased precipitation to measure changes in their traits (production, mass, germination). Seedlings germinated from those seeds were grown in a greenhouse to examine the effects of improved N and water availability in maternal and offspring environments on seedling growth. Maternal N-addition stimulated seed production, but it suppressed seed mass, germination rate and seedling biomass of *P. tanacetifolia*. Maternal N-addition also enhanced responses of seedlings to N and water addition in the offspring environment. Maternal increased-precipitation stimulated seed production, but it had no effect on seed mass and germination rate. Maternal increased-precipitation enhanced seedling growth when grown under similar conditions, whereas seedling responses to offspring N- and water-addition were suppressed by maternal increased-precipitation. Both offspring N-addition and increased-precipitation stimulated growth of seedlings germinated from seeds collected from the maternal control environment without either N or water addition. Our observations indicate that both maternal and offspring environments can influence seedling growth of *P. tanacetifolia* with consequent impacts on the future population dynamics of this species in the study area.

**Conclusion/Significance:**

The findings highlight the importance of the maternal effects on seed and seedling production as well as responses of offspring to changing environmental drivers in mechanistic understanding and projecting of plant population dynamics under global change.

## Introduction

Seeds as new offspring exert a dominant influence on the diversity and composition of plant community [1] by compensating for mortality of individual plants in a community and maintaining genetic variability of populations [2]. Changes in seed quantity and quality affect offspring number and growth and survival, with consequent influences on plant population dynamics and community structure [3]. In order to acclimate to varying environments, plants have to change their reproductive partitioning in terms of the number, size, and quality of seeds that they produce [4], [5]. According to the central tenet of life-history theory, there is a trade-off between number and size of offspring [6]. On the one hand, greater number of seeds ensures plants more opportunity to colonize new environments by generating more seedlings. On the other hand, larger seeds hold a larger fraction of scarce resources in reserve than smaller ones, contributing to a large part of the nutrient supply for the initial growth of seedlings [1]. Therefore, larger seeds, within a species in particular, are advantageous in seedling establishment, especially under unfavorable conditions [4].

Changing precipitation regimes [7], [8] and atmospheric N deposition [9], [10], [11] have profound impacts on plant community structure and biodiversity in the terrestrial biosphere [3], [12], [13], [14]. However, most previous studies focused on the effects of changing precipitation and N deposition on mature plants in terms of abundance, biomass and dominance. In contrast, little is known about how plant seeds and offspring respond to environmental changes and the possible subsequent influence on plant population dynamics and community composition. It has been proposed that plant populations respond to global change not only with plastic changes in phenotype, but also by altering offspring performance through transgenerational effects [5]. Therefore, elucidation of mechanisms underlying the changes in seeds and offspring attributes in response to climate change is urgently needed to predict and explain the shifts in species composition and biodiversity in terrestrial ecosystems.

Nitrogen and water are generally co-limiting factors that control plant growth and net primary productivity in terrestrial communities [15]. Nitrogen and water addition may pose positive impacts on maternal investment (in terms of seed production and seed mass) via stimulating biomass accumulation [16], [17], [18], [19], [20] and allocation of assimilate to reproduction [21], [22], [23], [24]. Nevertheless, reduced plant C/N ratio under N enrichment may lead to smaller seeds [25] according to the McGinley–Charnov hypothesis that optimal seed size positively depends upon the ratio of plant C to N pools available to a plant [26]. Changes in plant reproductive partitioning under improved N and water availability can substantially affect the quality and quantity of plant seeds and offspring. However, it remains unclear whether N addition and increased precipitation have additive or non-additive (interactive) effects on seeds and offspring of plants.

As part of a multi-factor field experiment initiated in April 2005 [27], [28], [29], [30], [31], this study was conducted to examine potential impacts of increased atmospheric N deposition and precipitation on seeds and seedlings of *P. tanacetifolia*, in a semiarid temperate steppe in northern China. Water and N availability are limiting factors for the steppe ecosystem in the area as evidenced by the marked stimulation of gross ecosystem primary productivity in response to water and N addition [32], [33], [34]. It is predicted that mean annual precipitation will increase by 30–100 mm in this century [35], whereas airborne N input is estimated to be 8–9 kg ha^−1^ yr^−1^ [36] and higher N deposition is expected in the area owing to land use change and anthropogenic activities [37]. Increased atmospheric N deposition, precipitation, and temperature have been documented to profoundly impact biodiversity and community composition in this steppe ecosystem [30], [31]. The specific objectives of this study were to reveal (1) how N addition, increased precipitation and their interaction affect seed production, mass, and germination rate of *P. tanacetifolia* and (2) whether maternal environment will affect offspring growth and their responses to N addition and increased precipitation.

## Methods

### Ethical statement

All work was undertaken with relevant permissions from the Restoration Ecology Research Station of CAS and Duolun County, Inner Mongolia, China for our observational and field studies.

### Study site

This study was conducted in a typical temperate steppe in Duolun County (42°02′N, 116°17′E, 1324 m asl), Inner Mongolia, China. Mean annual temperature is around 2.1°C with monthly mean extreme temperatures of 18.9°C in July and −17.5°C in January. Mean annual precipitation is about 385.5 mm with approximately 80% falling from June to September. The major soil type is chestnut (Chinese classification) or Calcic Luvisols according to the FAO classification system. The plant community at the experimental site is dominated by *Stipa krylovii* Roshev.(mean coverage approx. 5.8%), *Artemisia frigida* Willd. (12.7%), *Potentilla acaulis* L. (1.3%), *Potentilla tanacetifolia* Willd. (1.0%), *Cleistogenes squarrosa* (Trin.) King (0.7%), *Allium bidentatum* Fisc. ex prokh. (1.0%), and *Agropyron cristatum* (L.) Gaertn (2.1%). Canopy coverage of the community varies from 18.8% to 38.9% depending on year (Yang et al., unpublished data). Soil organic C and total N contents averaged 16.10±0.89 and 1.48±0.10 g kg^−1^, respectively.

*P. tanacetifolia*, a perennial forb, is a sparsely spread species, accounting for approximately 1.0% of the total coverage in the steppe community. It usually flowers in July and August. Seed production and dispersal take place in early September. The species proved to has extensive plasticity in terms of its morphology and physiology under different environmental conditions [38]. Owing to its high sensitivity to water and nutrient conditions as well as to livestock grazing, this species is usually regarded as an indicator plant of increasing habitat “quality” for community succession. In addition, *P. tanacetifolia* is more suitable for seed traits studies compared with the dominant species which regenerate or expand mainly via clonal and asexual reproduction in the community.

### Field experimental design

*In situ* observations were conducted at the permanent site of the Duolun Global Change Multifactor Experiment (GCME) established in 2005. The experiment employed a paired, nested design, with N addition manipulated at the plot level and precipitation manipulated at the subplot level. Four pairs of 44×28 m^2^ plots were set up, with one of each pair assigned as control (C) and another as N addition (N) in a random way. Nitrogen at 10 g N m^−2^ (urea in 2005 and NH_4_NO_3_ in 2006–2008) was applied once a year in middle July. In each control or N addition plot, two 10×15 m^2^ subplots were selected, and one was unwatered and the other was watered in summer (July and August). Fifteen millimeters of water was added once a week, leading to an annual addition of 120 mm precipitation (approximately 30% of the mean annual precipitation in the study site).

### Measurements of vegetation and seed attributes

In September 2007, one permanent 1×1 m^2^ quadrat was randomly selected in each plot or subplot, thus forming four replications for each treatment. The numbers of individual plants, vegetative tillers and reproductive tillers of *P. tanacetifolia* were recorded. Three reproductive tillers in each plot or subplot were sampled to calculate the fruit number. Three fruits in each plot or subplot were used to calculate the seed number per fruit. Seed production was calculated as the number of seeds per fruit×the number of fruits per reproductive tiller×the number of reproductive tillers per square meter. Mature seeds in each plot or subplot were collected and air-dried for use in the next growing season.

Seeds collected from the plots of each treatment in the field were mixed together. Five hundred mature seeds in each treatment were selected randomly to measure germination rate for each treatment. The 500 seeds were divided into 5 groups. Every100 seeds were weighed to determine the mean seed mass by an electronic balance with the accuracy of 0.1 mg, and were then placed on a moist filter paper in a sealed dish and kept at a constant temperature of 25°C in an incubating oven. Even though the germination test on moist filter paper cannot simulate the natural germination status of seeds in the field, the test avoided biased estimates on seed germination potential by excluding influence of environmental factors, i.e., heterogeneity of temperature, moisture, and light in the field. The cumulative seed germination rate was observed every day for 20 days until no more seeds germinated. Germination was determined by presence of the radicle. Seed attributes included germination rate and mass. Maternal investment was assessed in terms of seed production and seed mass. Seed quality was expressed by seed germination rate.

### Greenhouse observations

To examine the effects of maternal environment on offspring growth, a greenhouse adjacent to the field site was set up in the spring of 2008. Seeds collected from the field plots or subplots with the varying treatments in September 2007 were germinated in the greenhouse under the same conditions in late April of 2008. After germination, seedlings from each of the 4 maternal environments (control, N addition, increased precipitation, and N plus precipitation) were transplanted into pots (15 cm in diameter and 15 cm in depth) kept in the greenhouse in the middle of May. All the soil in the pots was collected from the field outside the treated plots. At the end of May, the seedlings together with pots were assigned to 4 offspring treatments including control, N addition, increased precipitation and N plus precipitation with seven replicates for each treatment according to a full-factorial design. Each pot was watered with 100 ml of water per day to maintain growth. Beginning in late May, watering was replaced with 100 ml of 0.5 g L^−1^ NH_4_NO_3_ solution added to the N-addition alone as well as to the N plus precipitation pots for 10 times at a 10-day interval during the growing season. The precipitation treatment was started at the beginning of August after full establishment of seedlings in the precipitation-increased-alone and in N plus precipitation pots. Given the sandy soil in the small container and higher temperature in the greenhouse, 200 ml of water per day was added to the precipitation-increased pots in order to make a substantial difference in water availability compared to the ambient watering treatments (100 ml water per day). Seven replicates were set for each treatment. Plants were harvested in late August, 2008 and oven-dried at 65°C for 48 h to constant weight for determination of biomass.

### Statistical analyses

ANOVAs for a split-plot design with N addition as the primary factor and water addition as the second factor were used to examine the main and interactive effects of maternal N-addition and increased-precipitation on seed production. Because seeds were mixed at treatment level after seed production measurement, it was impossible to distinguish which plot or subplot they came from. Thus two-way ANOVAs with a full factorial design were used to examine the main and interactive effects of maternal N-addition and increased-precipitation on seed mass, germination, seedling biomass, and seedling responses to the offspring treatment. Two-way ANOVAs with full factorial design were also used to address the impacts of offspring treatments on seedling biomass. Linear regressions were used to examine the relationships of seed mass with seed production and seed germination rate with seed mass. All the above statistical analyses were conducted with SAS software (SAS Institute Inc., Cary, NC, USA).

## Results

### Seed production, weight, germination rate and their interdependence

**Figure 1:**
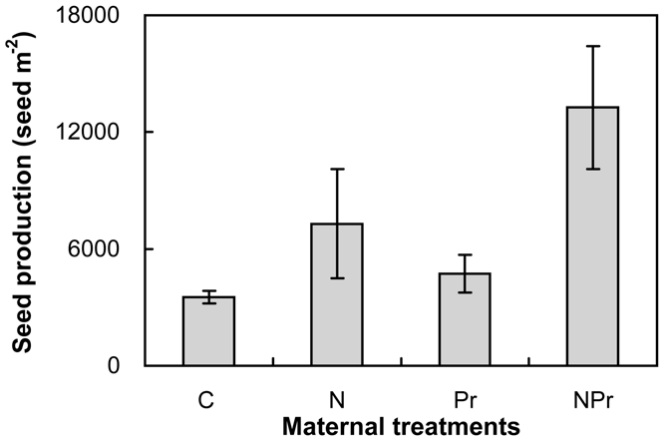
Effects of maternal-treatments on seed production of *Potentilla tanacetifolia.* Data are mean ± SE (n = 4). C: control treatment; N: N addition; Pr: increased precipitation; NPr: N addition plus increased precipitation.

N-addition in the maternal environment increased seed production of *P. tanacetifolia* by 148.7% (*P<*0.001; ANOVAs with split design) across the 2 maternal precipitation treatments. Seed production was enhanced by 66.4% (*P<*0.05) under maternal increased-precipitation treatment. Maternal N-addition and increased-precipitation also interacted to affect seed production (*P<*0.05, Fig. 1). Maternal N-addition stimulated seed production by 106.7% and 180.0% under the maternal ambient- (N versus C) and increased-precipitation (NPr versus Pr), respectively. Maternal increased-precipitation enhanced seed production by 34.3% without maternal N-addition (Pr versus C), but it reduced seed production by 81.9% with maternal N-addition (NPr versus N).

**Figure 2:**
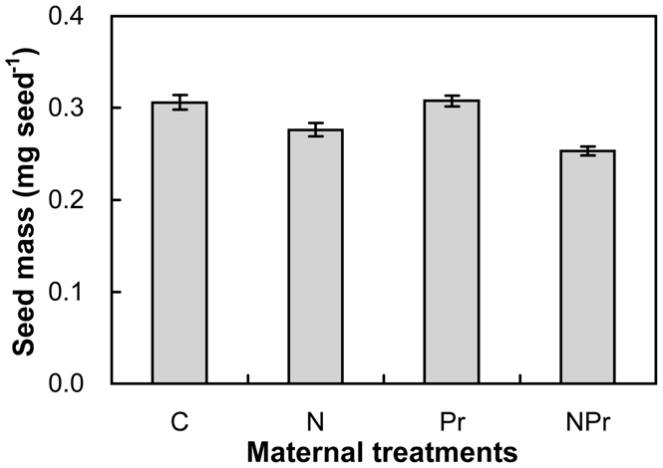
Effects of maternal-treatments on seed mass (mg seed^−1^) of *Potentilla tanacetifolia*. Data are mean ±SE (n = 5). See Figure 1 for abbreviations.

Maternal N-addition significantly decreased seed mass, on average, by 16.1% (*P<*0.01; two-way ANOVAs) across the 2 maternal precipitation regimes, whereas maternal increased-precipitation had no effect on seed mass (*P*>0.10). There was a marginally interactive effect on seed mass (*P*<0.10). Maternal N-addition suppressed seed mass by 9.7% and 17.7% under the maternal ambient- (N versus C) and increased-precipitation (NPr versus Pr), respectively. Maternal increased-precipitation stimulated seed mass by 0.5% without maternal N-addition (Pr versus C), but it reduced seed mass by 8.3% with maternal N-addition (NPr versus N, Fig. 2).

**Figure 3:**
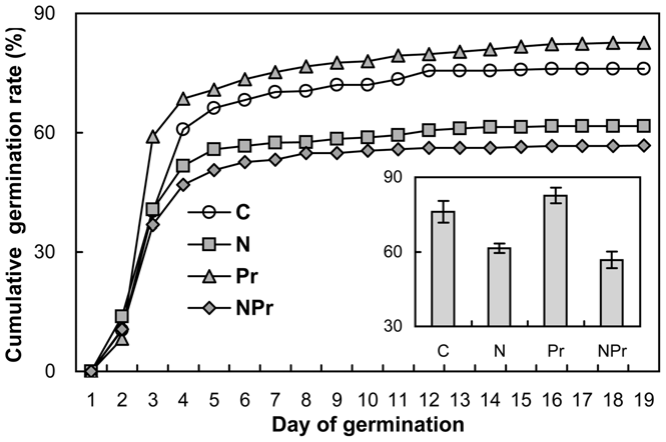
Effects of maternal-treatments on seed cumulative germination rate (%) and final germination percentage (insets, means ±1SE, n = 5). The seeds were collected from the plots with 4 maternal treatments in the field and germinated under the same conditions in the greenhouse. See Figure 1 for abbreviations.

Maternal N-addition decreased seed germination rate, on average, by 20.1% (absolute difference, *P<*0.01) across the 2 maternal precipitation treatments. Maternal increased-precipitation had no main effect on seed germination rate (*P*>0.10). There was no interactive effect of maternal N-addition and increased-precipitation on seed germination (*P*>0.10, Fig. 3).

**Figure 4:**
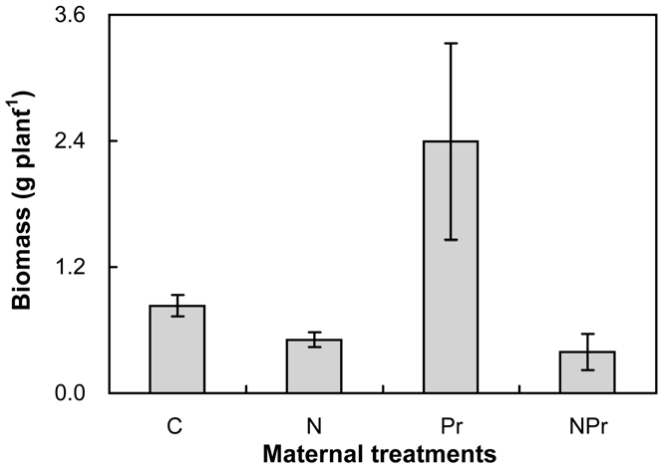
Dependence of seed mass upon seed production (Y = −5.84^−06^X+0.3278) and germination percentage upon seed mass (Y = 441.95X−57.04) across all the 4 maternal treatments. Each maternal treatment was marked in the panel. See Figure 1 for abbreviations.

Across the 4 maternal treatments, seed mass showed a strong negative dependence upon seed production (r^2^ = 0.925, P<0.05, Fig. 4a). In contrast, seed germination rate linearly increased with seed mass (r^2^ = 0.926, P<0.05, Fig. 4b).

### Seedling Biomass

**Figure 5:**
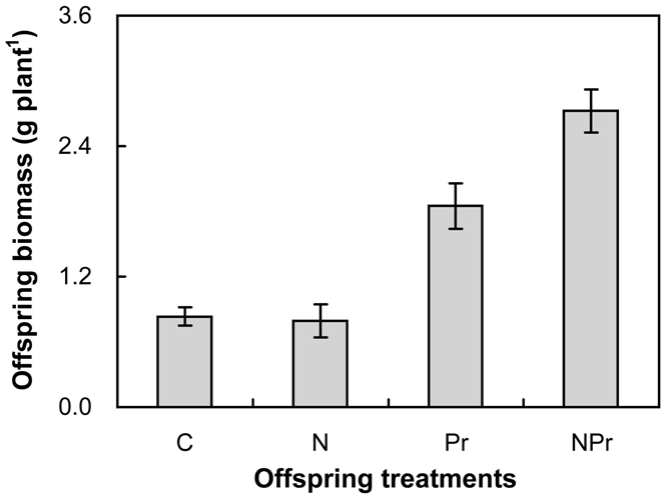
Effects of maternal-treatments on seedling biomass (g). The seedlings were germinated from the seeds collected from the plots with 4 maternal treatments in the field and grown under the same offspring environment in the greenhouse. Data are mean ±SE (n = 7). See Figure 1 for abbreviations.

Maternal N-addition significantly reduced seedling biomass, on average, by 97.2% (*P<*0.01, Fig. 5) across the 2 maternal precipitation regimes. In contrast, maternal increased-precipitation significantly stimulated seedling biomass, on average, by 107.5% (*P<*0.05) across the 2 maternal N levels. Maternal N-addition and increased-precipitation also interacted with each other to affect seedling biomass (*P<*0.05). Maternal N-addition decreased seedling biomass by 39.0% and 83.6% under the maternal ambient- (N versus C) and increased-precipitation (NPr versus Pr), respectively. Maternal increased-precipitation enhanced seedling biomass by 187.0% without maternal N-addition (Pr versus C). In contrast, it suppressed seedling biomass by 22.8% with maternal N-addition (NPr versus N, Fig. 5).

**Figure 6:**
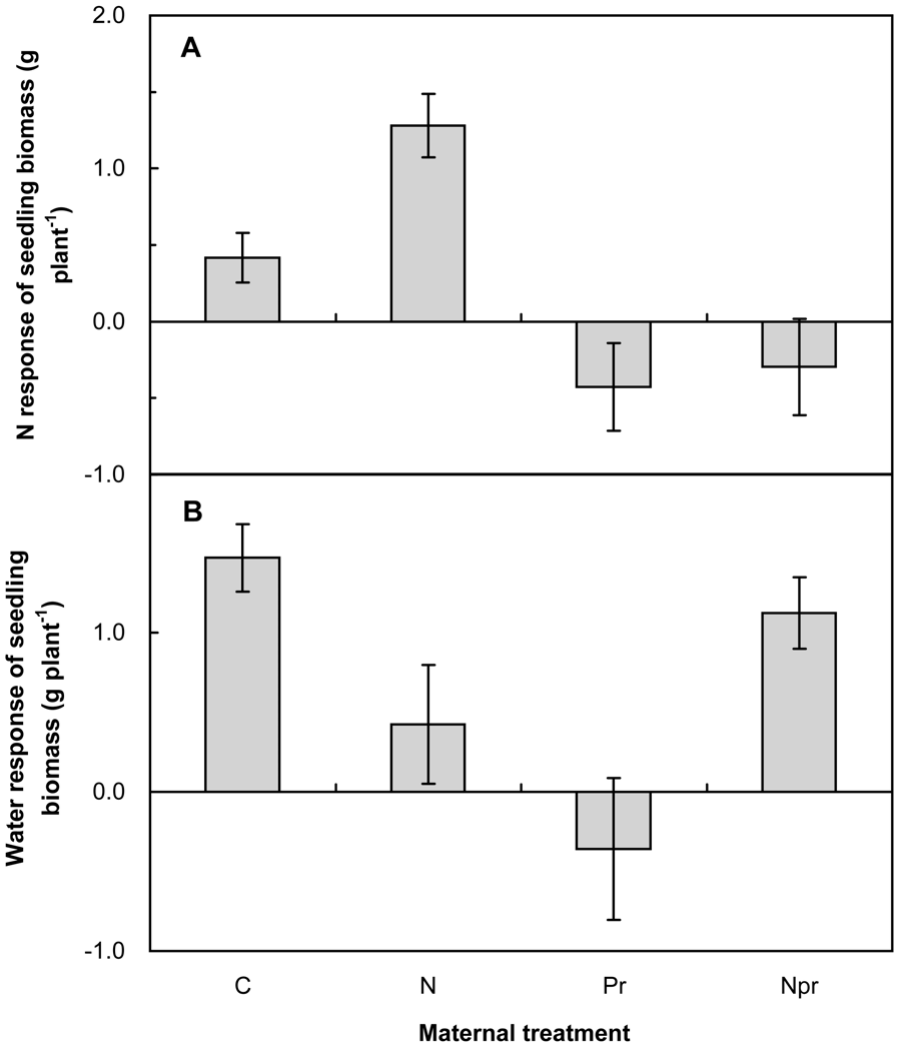
Effects of maternal-treatments on the N (a) and water (b) responses of seedling biomass. Data are mean±SE (n = 7). See Figure 1 for abbreviations. The seedlings were germinated by the seeds collected from the 4 maternal treatment plots (control, N addition, increased precipitation, and N addition plus increased precipitation) in the field and grown under the 4 offspring treatments (control, N addition, increased precipitation, and N addition plus increased precipitation) in the greenhouse. The N responses were calculated as the absolute difference (g plant^−1^) in seedling biomass between with and without offspring N-addition. The water responses were calculated as the absolute difference in seedling biomass between the offspring ambient- and increased-precipitation.

Both maternal N-addition environment and maternal increased-precipitation environment had significant main effects on responses of the seedling to changing N and water status in the offspring environment. The seedling responses to offspring N-addition were changed from negative (−0.0048 g plant^−1^) without maternal N-addition to positive (+0.4915 g plant^−1^, *P<*0.10) with maternal N-addition (Fig. 6a). In contrast, maternal increased-precipitation significantly shifted the seedling responses to offspring N-addition from positive (+0.85 g plant^−1^) to negative (−0.36 g plant^−1^, *P<*0.001). Maternal N-addition and increased-precipitation did not interact to affect the N response of seedlings (*P>*0.10). In conclusion, if the maternal environment experienced increased-precipitation, response to offspring N-addition was negative; if the maternal environment experienced ambient precipitation, the response to offspring N-addition was positive.

Maternal N-addition slightly, but insignificantly, increased the seedling response to offspring increased-precipitation, on average, by 39.5% (*P>*0.10) across the 2 maternal precipitation regimes. On the contrary, maternal increased-precipitation marginally reduced the responses of seedlings to the water by 59.6% (*P<*0. 10, Fig. 6b) across the 2 maternal N treatments. Interaction of maternal N-addition and increased-precipitation significantly affected the response of seedlings to water (*P*<0.001). Maternal N-addition decreased the response of seedlings to water by 71.2% and 413.6% under the maternal ambient- (N versus C) and increased-precipitation (NPr versus Pr), respectively. Maternal increased-precipitation suppressed the response of seedlings to water by 124.4% without maternal N-addition (Pr versus C), but stimulated it by 165.2% with maternal N-addition (NPr versus N, Fig. 6b). In conclusion, if the maternal environment only experienced increased-precipitation, the response to offspring increased-precipitation was negative; if the maternal environment experienced N-addition, regardless of water availability, the response to offspring increased-precipitation was positive. Thus, from a maternal environment standpoint, increased-precipitation alone always had negative effects; N-addition alone always had positive effects; but the response to the joint treatment of N-addition and increased-precipitation depended on the offspring environment (negative in response to N, positive in response to water).

**Figure 7:**
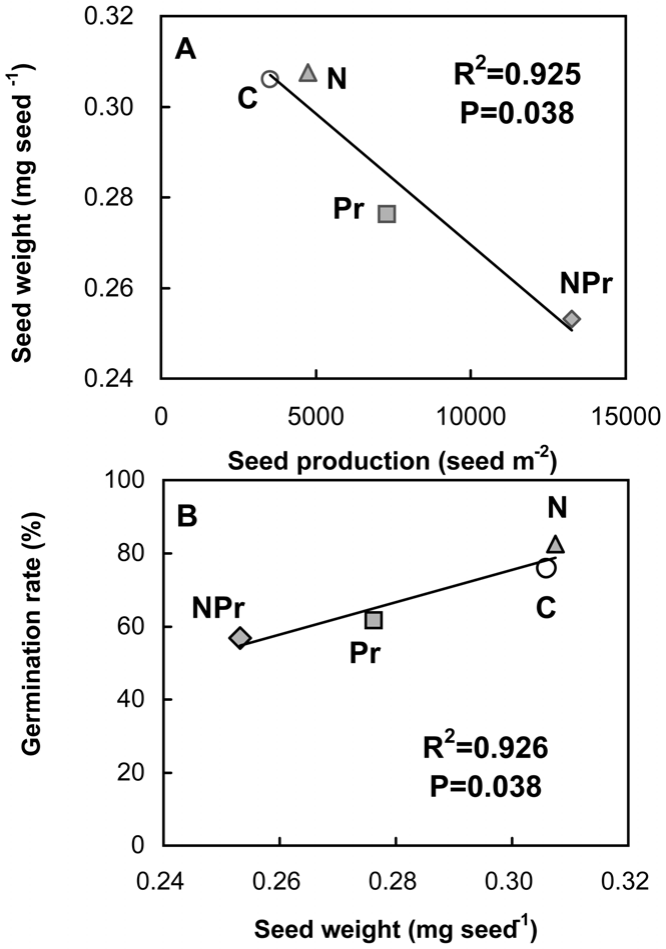
Effects of offspring-treatments on seedling biomass (g). The seedlings were germinated by seeds collected from the maternal control treatment. Data are mean ± SE (n = 7). See Figure 1 for abbreviations.

All offspring N-addition (*P*<0.05), increased-precipitation (*P*<0.01) and their interactions (*P*<0.05) had significant influence on the seedling growth. The seedlings were germinated by seeds collected from the maternal plants in the control environment (without addition of either N or water). Offspring N-addition significantly stimulated seedling biomass, on average, by 34.6% across the 2 offspring precipitation regimes. Seedling biomass under offspring ambient-precipitation was, on average, 181.4% greater than that under offspring increased-precipitation (Fig. 7). Offspring N-addition reduced seedling biomass by 4.8% under the offspring ambient-precipitation (N versus C), but it enhanced seedling biomass by 47.3% under offspring increased-precipitation (NPr versus Pr). Offspring increased-precipitation stimulated seedling biomass by 121.8% and 243.0% without (Pr versus C) and with offspring N-addition (NPr versus N), respectively.

## Discussion

Changing global environment is predicted to affect population dynamics of terrestrial plants, with consequent influences on community composition and biodiversity in the terrestrial biosphere [39]. On the one hand, maternal environment affects seed traits, such as seed production and seed mass, which influence population dynamics and are thus responsive to environmental change.. On the other hand, environmental change may potentially impact plant population dynamics via its transgenerational effects on plants' growth and their responses to the driving environmental factors [5]. With both field and greenhouse experimental manipulations, our study indicates that it is the offspring growth that is affected.

### Impacts of maternal environment on seed production, weight and germination rate

Enhanced N environment experienced by the maternal plants undoubtedly plays an important role in determining phenotypic variation in seeds of *P. tanacetifolia* in our study. Our observations are in line with those reported in the previous studies at a desert [3] and a prairie-forest border [39]. N addition usually stimulates total plant biomass and resource allocation to reproductive organs [21], [23]. This in turn leads to an increase in maternal plant height [23], [40] and a decrease in abortion of flowers and fruits [41], [42]. Larger plants often produce bigger or greater number of seeds if allocation of resource to reproductive structures is increased as plants increase in size [43],[44]. Reproductive biomass influences the role that maternal environment plays in seed production. In this study, N addition stimulated maternal plant reproductive biomass by 25.7% in *P. tanacetifolia* (Li Y, unpublished data) and subsequently to enhanced seed production (Fig. 1). Given a certain amount of reproductive allocation and the trade-off between seed mass and production [6], greater number of seeds would favor generation of smaller seeds. Our observations that seed mass was strongly negative dependent upon seed production across the 4 treatments (Fig. 4a) support this speculation. In addition, the smaller seeds may also be accounted for by the reduced plant C/N ratio under N enrichment (Fig. 2) according to McGinley–Charnov hypothesis [26].

More seeds were produced but they were small. On the one hand, more seeds produced increase the probability that a seed will find a safe site. In order to colonize and establish, plants have to allocate the majority of their resources to seeds in habitats with fluctuating environmental conditions [39]. Higher seed production makes it more probable for a species to be successful in reproduction [45] even if seed predators consume more seeds. On the other hand, smaller seeds have less energy reserves and thus have poor germination, because all nutrients needed during embryo development come from maternal investment [46]. The influence of maternal N-addition on seed germination could therefore operate through seed mass. The positive dependence of germination rate upon seed mass of *P. tanacetifolia* across the 4 treatments in the maternal environment (Fig. 7b) was observed in this study.

Our results demonstrate that increased precipitation in the maternal environment stimulated seed production, but it had no detectable influence on seed mass or germination rate of *P. tanacetifolia* growing in the semiarid region. Water is a major limiting factor for plants' growth and establishment in this ecosystem, and an increase in water availability is expected to stimulate plant growth and reproduction. In this study, the maternal increased-precipitation was conducted in July and August every year when *P. tanacetifolia* had flowered and began to produce seeds. This would lead to enhanced seed production (Fig. 1). However, water is a resource that seeds cannot reserve [1], thus the increased water availability under the maternal increased-precipitation had no impacts on seed mass and germination rate. The marginally interactive effect of maternal increased-precipitation with N-addition on seed mass suggests that improved water availability may indirectly promote N translocation by nutrient availability to the developing seed [47], [48].

### Impacts of maternal environment on seedling growth and their response to changing offspring environment

The significant effects of maternal environments on seedling biomass may be mediated by seed mass, because the seedlings germinated from larger seeds perform better owing to their greater metabolic reserves [49], [50], [51], [52], [53], [54], [55]. Seedlings make use of nutrients contained in seeds until radicles can absorb nutrient from soil [56]. Thus the bigger seed would allow radicles to quickly break seed coat, facilitating absorption of nutrient from soil [46], [54]. Earlier germinating seeds may produce more surviving seedlings than later germinating seeds under competitive environment [57], because the earlier germinating seeds may have an advantage over the later germinating seeds to soil and light resources. Therefore, the seedlings germinated from bigger seeds can have more biomass. In this study, we found that reduced seed mass (Fig. 2) under the maternal N-addition resulted in lower seedling biomass of *P. tanacetifolia* (Fig. 5), implying that transgenerational effects on plants of N enrichment did occur.

Since summer precipitation and N deposition in northern China are predicted to increase in the future [58], knowledge of how these factors will impact plant responses in this area is urgently needed. Offspring responses to varying environmental changes may be explained by the effects of both maternal and offspring environments. Through transgenerational effects on plasticity [5], maternal environments can not only influence seedling growth, but also impact the seedling responses to varied environments. Our observations illustrate that maternal N-addition negatively affected seedling biomass of *P. tanacetifolia*, whereas it changed the response of seedlings to offspring N-addition. On the contrary, maternal increased precipitation stimulated seedling biomass, but it suppressed the responses of seedlings to water in the offspring environment. The contradictory effects make it difficult to evaluate the net maternal effects on the growth, competition, and establishment of *P. tanacetifolia* offspring in plant communities.

Offspring environment may be more important for offspring growth and survival than the maternal environment [4], [59], [60] because plants lack the ability to escape from severe environment. When *P. tanacetifolia* seedlings germinated from seeds collected from the maternal control plots were treated with N addition and/or increased precipitation, both offspring N-addition and increased-precipitation enhanced seedling biomass of *P. tanacetifolia*. However, the effects of maternal and offspring environments may counteract each other such that maternal and offspring N-addition reduced and stimulated seedling biomass, respectively. The positive effects of both maternal and offspring increased-precipitation on seedling biomass could be counterbalanced by the negative impacts of the maternal increased-precipitation on the water responses of offspring. Furthermore, the interactive effects of increased N and precipitation on seedling biomass depended upon maternal and offspring environments. We showed diverse changes in seedling biomass and responses of seedlings to offspring environment when parents experienced maternal N-addition and/or increased-precipitation treatments. These observations indicate uncertain population dynamics of *P. tanacetifolia* under future N deposition and changing precipitation regimes. In fact, no significant changes in biomass, coverage, or abundance of *P. tanacetifolia* were observed in our 6 year field survey from 2005–2010 (Yang *et al*. personal communication).

Global change may also see rising temperatures and CO_2_ concentrations. Effects of maternal-warming on seeds and seedlings could take place via altering water availability. According to a meta-analysis, CO_2_ enrichment also posed substantial effects on seed production and seed mass [59] for most species [61], [62]. In addition, it seems that some species were more responsive to elevated CO_2_ than were others. These differences would have broad implications for the species composition and functioning of future ecosystems.

### Conclusions

As a non-clonal species, the regeneration of *P. tanacetifolia* in plant communities largely depends on seeds in the semi-arid temperate steppe of northern China. Changes in seeds and seedlings traits would affect its reproductive success and population dynamics. Our results demonstrate that maternal N-addition significantly affected seed production, seed mass, germination rate, offspring biomass and their responses to offspring N-addition. Maternal increased-precipitation had significant effect on offspring biomass and their responses.to water regimes. Both offspring N-addition and increased-precipitation enhanced seedling biomass. However, the diverse responses of the seed and seedling traits of *P. tanacetifolia* to N addition, increased precipitation and their interactions in both maternal and offspring environments make it difficult to predict the future population dynamics of this species under increased atmospheric N deposition and changing precipitation regimes.
